# Acetylation of glucokinase regulatory protein decreases glucose metabolism by suppressing glucokinase activity

**DOI:** 10.1038/srep17395

**Published:** 2015-12-01

**Authors:** Joo-Man Park, Tae-Hyun Kim, Seong-Ho Jo, Mi-Young Kim, Yong-Ho Ahn

**Affiliations:** 1 Department of Biochemistry and Molecular Biology, Yonsei University College of Medicine, 50-1 Yonsei-ro, Seodaemun-gu, Seoul 120-752, Republic of Korea; 2 Brain Korea 21 PLUS Project for Medical Sciences, Yonsei University College of Medicine, 50-1 Yonsei-ro, Seodaemun-gu, Seoul 120-752, Republic of Korea

## Abstract

Glucokinase (GK), mainly expressed in the liver and pancreatic β-cells, is critical for maintaining glucose homeostasis. GK expression and kinase activity, respectively, are both modulated at the transcriptional and post-translational levels. Post-translationally, GK is regulated by binding the glucokinase regulatory protein (GKRP), resulting in GK retention in the nucleus and its inability to participate in cytosolic glycolysis. Although hepatic GKRP is known to be regulated by allosteric mechanisms, the precise details of modulation of GKRP activity, by post-translational modification, are not well known. Here, we demonstrate that GKRP is acetylated at Lys5 by the acetyltransferase p300. Acetylated GKRP is resistant to degradation by the ubiquitin-dependent proteasome pathway, suggesting that acetylation increases GKRP stability and binding to GK, further inhibiting GK nuclear export. Deacetylation of GKRP is effected by the NAD^+^-dependent, class III histone deacetylase SIRT2, which is inhibited by nicotinamide. Moreover, the livers of *db/db* obese, diabetic mice also show elevated GKRP acetylation, suggesting a broader, critical role in regulating blood glucose. Given that acetylated GKRP may affiliate with type-2 diabetes mellitus (T2DM), understanding the mechanism of GKRP acetylation in the liver could reveal novel targets within the GK-GKRP pathway, for treating T2DM and other metabolic pathologies.

The worldwide incidence of type 2 diabetes mellitus (T2DM) is increasing, due to the rising adoption of western-style diets and sedentary lifestyles^1^. A universal feature of T2DM is increased blood glucose^2^, the primary cellular energy source that must normally be maintained at approximately 5 mM by various organs. Among these, the liver is crucial to glucose homeostasis, by controlling glucose import and export, depending on dietary and metabolic needs throughout the body.

Glucokinase (GK; ATP: D-hexose 6-phosphotransferase, hexokinase-4), an enzyme mainly expressed in liver and pancreatic β-cells, is pivotal to maintaining homeostatic blood glucose levels^3^,^4^. Thus, understanding the mechanisms governing GK activity and expression is essential for developing therapeutics for T2DM and other metabolic disorders. GK converts glucose to glucose-6-phosphate by transferring a phosphate group from ATP to glucose, the first step in glycolysis and glycogenesis^5^. The GK-encoding gene *GCK* is regulated in a tissue-specific manner, due to the presence of alternative upstream β-cell- and downstream liver-specific promoters^6^. Liver *GCK* is mainly upregulated by insulin, an effect opposed by glucagon^6^,^7^. Binding sites for transcription factors, including those for sterol regulatory element-binding protein-1c (SREBP-1c), liver X receptor alpha (LXRα), hypoxia inducible factor-1 alpha (HIF-1α), and insulin-like growth factor-1 (IGF-1), are all present within the *GCK* promoter^8^,^9^, thus demonstrating its intricate response to a myriad of physiological conditions (*e.g*., hypoxia, hormone levels, metabolic stress, *etc*.).

Other regulatory molecules, such as phosphate esters^10^, are crucial modulators of GK activity that bind the glucokinase regulatory protein (GKRP)^11^. GKRP is normally compartmentalized in the nucleus, and its relative instability frees GK to shuttle between the nucleus and the cytosol in response to metabolic status^12^,^13^. The interaction between these two proteins is promoted by binding of fructose-6-phosphate to GKRP, whereas fructose-1-phosphate weakens the interaction^11^,^14^. In addition, GKRP regulates the stability of GK, as shown in the GKRP knockout (KO) mouse, which has decreased GK protein levels and kinase activity^15^. GKRP is also regulated by phosphorylation by the AMP-activated protein kinase (AMPK), a master “energy sensor” that regulates glucose uptake and lipid biosynthesis; AMPK also inhibits nuclear GK efflux to the cytosol^16^. Thus, GKRP is essential to glucose homeostasis, warranting further study of its regulation by post-translational modifications (PTMs) such as phosphorylation, acetylation, glycosylation, and ubiquitination that all affect protein stability, intracellular compartmentalization, activity, and interaction with other proteins^17^.

In this study, we demonstrate that GKRP acetylation, by the acetyltransferase p300, represents a unique mechanism in the regulation of GK activity. We also show that GKRP acetylation decreases its ubiquitination and increases its affinity for GK, resulting in increased nuclear retention of GK and decreased glycolytic flux. Finally, we observed elevated GKRP acetylation in *db/db* (leptin receptor-lacking) mice, strongly suggesting a role for GKRP in T2DM and possibly, obesity.

## Results

### GKRP is prominently acetylated at lysine 5 by p300

In humans, GKRP expression is highest in the liver^10^, and we first examined GKRP mRNA in tissues from C57B/6J mice. While GKRP mRNA levels were (expectedly) highest in the liver, unlike humans, mouse white adipose tissue also expressed considerable GKRP mRNA (Supplementary Fig. S1).

**Figure 1:**
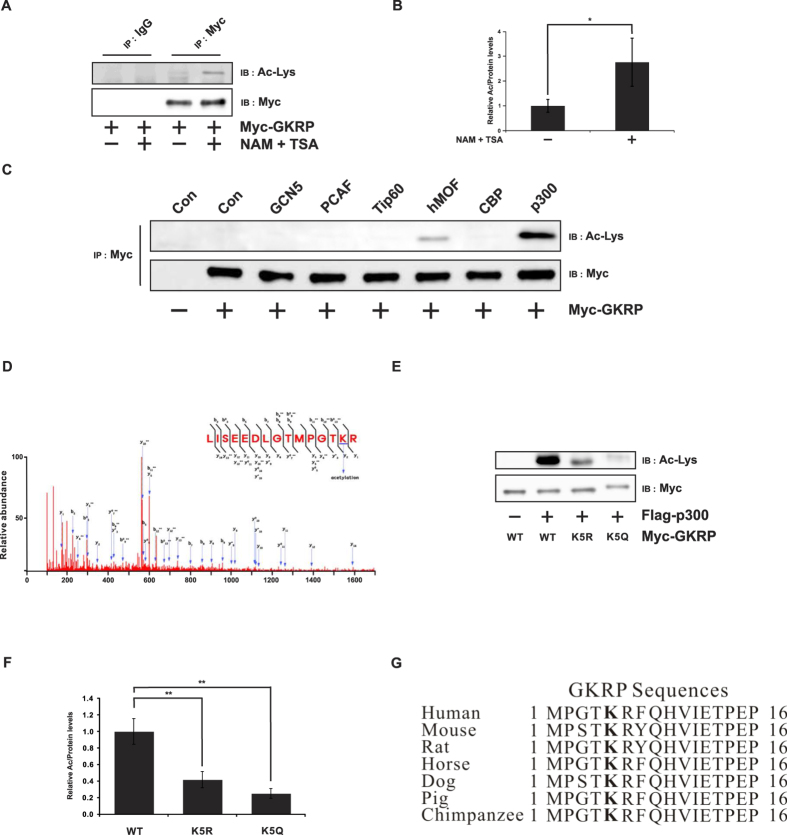
GKRP is acetylated by p300. (**A**) Effects of histone deacetylase inhibitors on GKRP. HeLa cells transfected with Myc-tagged GKRP were treated with 5 mM NAM and 1 μM TSA 6 hr before harvest. (**B**) Band intensities of acetylated Myc-GKRP were quantified by Image J software. The values from samples not treated with NAM and TSA were set to 1.0. Data are shown as the means ± SEM of four independent experiments. (**C**) Identification of the acetyltransferase responsible for GKRP acetylation. Expression vectors of various acetyltransferases (ATs) were co-transfected with pSG-Myc GKRP into HeLa cells. The immunoprecipitates (from antibodies against the various ATs) were then subjected to immunoblot with antibodies against Ac-Lys or Myc. (**D**) LC-MS/MS spectrum of GKRP peptides showing that acetylation occurs at K5. (**E**) Effects of site-specific mutation on the potential acetylation site, GKRP K5. Substitutions of Lys (K) with Arg (R) or Glu (Q) at the indicated sites are shown in parenthesis. HeLa cells transfected with the indicated mutant or wild-type plasmids were lysed and immunoprecipitated by an anti-Myc antibody. Acetylated GKRP was detected by an anti-Ac-Lys antibody. (**F**) Sequence alignment of the GKRP region containing K5 from various species. NAM, nicotinamide; TSA, Trichostatin A. Data are expressed as means ± SEMs, n = 4, *p ≤ 0.05; **p ≤ 0.01.

Since most metabolic enzymes are acetylated^18^, we assessed GKRP for possible acetylation that might modulate GK activity, in HeLa cells, which do not express GKRP, transfected with a Myc-GKRP fusion expression vector. Treatment with the histone deacetylase inhibitors (HDACIs) nicotinamide (NAM) and Trichostatin A (TSA)^19^ notably increased GKRP acetylation (Fig. 1A,B, p ≤ 0.05). To identify the acetyltransferase(s) responsible for GKRP acetylation, HeLa cells were cotransfected with expression vectors for Myc-GKRP and various acetyltransferases, including the General CoNtrol of amino synthesis (GCN5, KAT2), p300/CBP-associated factor (PCAF, KAT2B), HIV-1 Tat interactive protein 60 kDa (Tip60), human MYST histone acetyltransferase 1 (hMOF, KAT8), CREB-binding protein (CBP, CREBB2), or p300 (EP300). As shown in Fig. 1C, GKRP was predominantly acetylated by p300, followed by hMOF, in a dose-dependent manner (Supplementary Fig. S2A). Moreover, p300 and GKRP directly interacted with each other, as shown by co-immunoprecipitation (Supplementary Fig. S2B). To test whether p300 plays a role in regulating GKRP acetylation, we used C646, a p300-specific inhibitor^20^, to treat HeLa cells transfected with expression vectors for Myc-GKRP and Flag-p300. That assessment demonstrated that C646 treatment decreased GKRP acetylation (Supplementary Fig. S2C), indicating that p300 acetylates GKRP.

To determine the possible site(s) of GKRP acetylation, Prediction of Acetylation on Internal Lysines (PAIL) software (http://bdmpail.biocuckoo.org) revealed three potential N-terminal acetylation sites, Lys5 (K5), Lys170 (K170), and Lys261 (K261), all conserved among human, mouse, and rat GKRP protein sequences (see Supplementary Table S2)^21^. To narrow down the exact acetylation site(s), these three Lys residues in Myc-tagged GKRP were mutated to arginine (Arg, R, and GKRP deacetyl-mimic) or glutamine (Gln, Q, and GKRP acetyl-mimic). The K5R mutation most distinctly reduced overall GKRP acetylation, compared to the K170R or K261R mutants (Supplementary Fig. S2D). These results were further confirmed by LC-MS/MS, revealing K5 as GKRP’s major acetylation site (Fig. 1D). To further validate these results, cells were transfected with wild-type (WT) GKRP and two GKRP K5 mutants described above (K5R or K5Q). Those assessments showed that GKRP acetylation was significantly decreased by substituting an Arg residue for the highly conserved GKRP K5 (Fig. 1E and F, p ≤ 0.01) (Fig. 1G).

**Figure 2:**
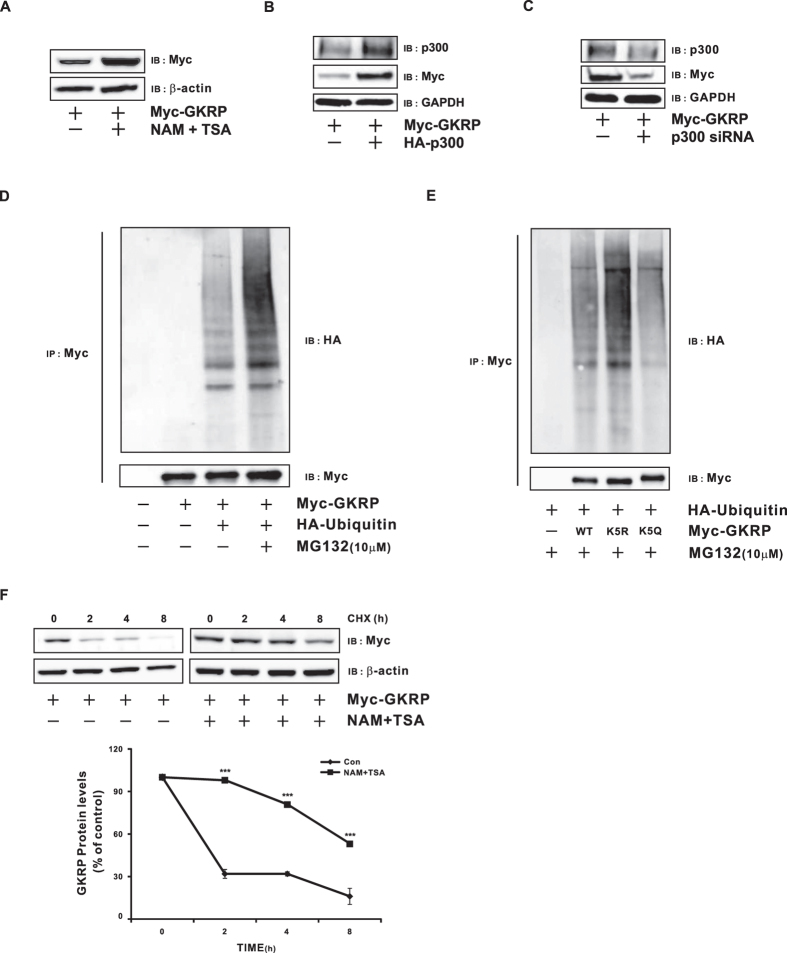
Ubiquitin-dependent GKRP degradation is decreased by acetylation. (**A**) Effects of histone deacetylase inhibitors on GKRP levels in. HeLa cells transfected with Myc-tagged GKRP. (**B**) Effect of p300 on GKRP protein levels in HeLa cells transfected with Myc-tagged GKRP and full-length p300. (**C**) Effect of anti-p300 siRNA on GKRP protein levels. HeLa cells transfected with Myc-tagged GKRP and negative control or p300 siRNA (10 nM) were incubated for 48 hr, and GKRP protein levels assessed by immunoblot using an anti-Myc antibody. (**D**) Effect of ubiquitin on GKRP stability. HeLa cells cotransfected with Myc-tagged GKRP and HA-tagged ubiquitin were maintained in the absence or presence of MG132 (10 μM) for 2 hr. Cell lysates were precipitated with an anti-Myc antibody and immunoblotting used to detect ubquitinated GKRP by anti-HA antibody. (**E**) Effect of various K5 mutants on GKRP ubiquitination. Myc-tagged wild type, deacetyl-mimic (K5R), or acetyl-mimic (K5Q) GKRP was cotransfected with HA-ubiquitin in the presence of the proteasome inhibitor MG132 (10 μM) for 2 hr. Cell lysates were precipitated by an anti-Myc antibody, and immunoblot with an anti-HA antibody used to detect ubiquitinated GKRP. (**F**) Effects of HDACIs on GKRP stability. HeLa cells were transfected with Myc-tagged GKRP were treated with NAM (5 mM) and TSA (1 μM) 4 hr before treating CHX. 24 hr after transfection, cells were treated with 50 μg/ml of the protein synthesis inhibitor CHX for the indicated time periods. GKRP protein levels from cells collected at time zero were defined as 100%. GAPDH and β-actin were used as an internal control. GAPDH, glyceraldehyde 3-phosphate dehydrogenase. HDACIs, histone deacetylase inhibitor, NAM, nicotinamide, TSA, Trichostatin A, and CHX, cycloheximide. Data are expressed as means ± SEMs, n = 4, ***p ≤ 0.001.

Acetylated GKRP acetylation resists ubiquitin-mediated degradation. To assess the effect of acetylation on GKRP stability, we treated HeLa cells with the aforementioned HDACIs, NAM and TSA, resulting in increased acetylated GKRP protein levels (Fig. 2A). Conversely, immunoblotting demonstrated that Myc-GKRP coexpression with HA-tagged full-length p300 increased, and anti-p300 siRNA decreased, GKRP protein levels (Fig. 2B,C). These results strongly suggest that GKRP acetylation by p300 might mediate its stability.

To explore regulation of GKRP stability by ubiquitin-dependent degradation, GKRP was coexpressed with HA-tagged ubiquitin in the absence or presence of MG132, a proteasome inhibitor. That assay demonstrated that downregulated proteasome activity substantially elevated ubiquitin-conjugated GKRP (Fig. 2D). To further confirm whether K5 acetylation increases GKRP stability, HA-tagged ubiquitin was co-expressed with WT or each of the two GKRP K5 mutants (K5R and K5Q), in the presence of MG132. Those specific combinations showed that indeed, GKRP ubiquitination increased in the K5R mutant, which mimics the deacetylated state, compared to the WT or the K5Q mutant, which mimics acetylated GKRP (Fig. 2E). Furthermore, GKRP degradation after cycloheximide (CHX, an inhibitor of protein biosynthesis) treatment showed that about 60% of the total GKRP protein remained after 8-hr HDACI (NAM and TSA) treatment, while GKRP degraded completely, after 4 h, in the absence of those HDACIs (Fig. 2F, p ≤ 0.001). These results support the conjecture that GKRP acetylation by p300 protects it from ubiquitin-dependent proteasomal degradation.

**Figure 3:**
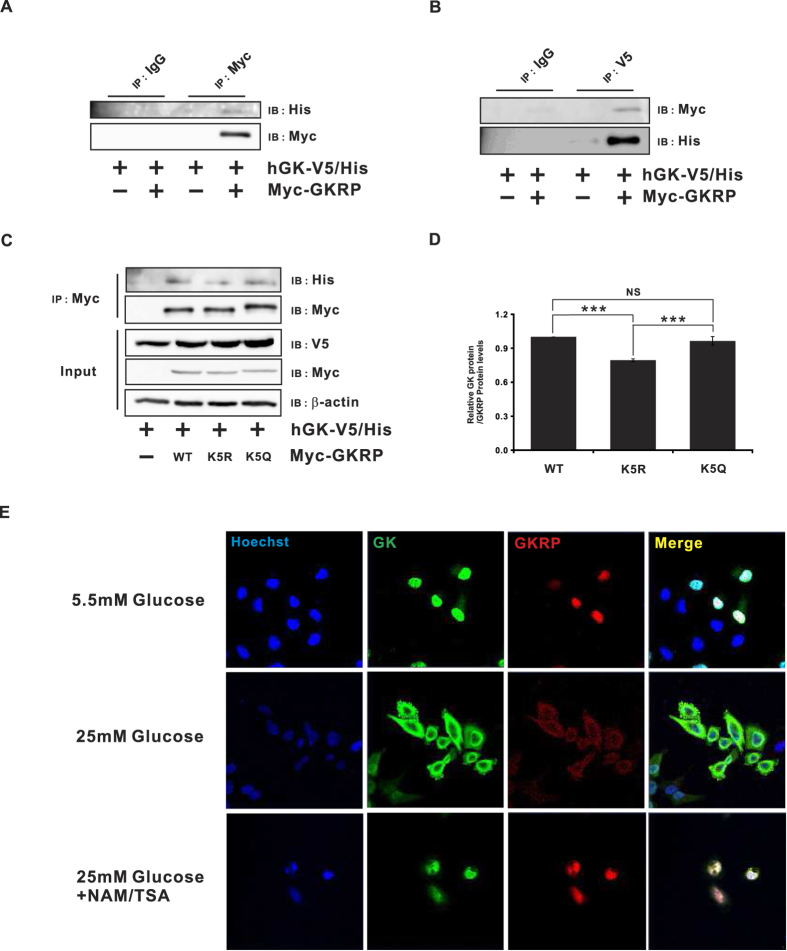
GKRP acetylation is critical for binding and retaining GK in the nucleus. (**A,B**) Interaction between GKRP and human glucokinase (hGK). HeLa cells transfected with expression vectors for GKRP and hGK were lysed, protein immunoprecipitated with anti-Myc or anti-V5 antibodies, and immunoblotted by anti-His or anti-Myc antibodies. (**C**) Effect of K5 mutations on GKRP interaction with hGK. HeLa cells cotransfected with hGK and wild type or the indicated GKRP mutants were lysed, protein precipitated with an anti-Myc antibody, and blotted with an anti-His antibody. β-actin expression was used as internal control. (**D**) Immunofluorescence micrographs showing the subcellular locations of GKRP and hGK. HeLa cells transfected with Myc-tagged GKRP and V5/His-tagged GK were maintained in 5.5 mM or 25 mM glucose for 4 hr in the absence (−) or presence (+) of a mixture of the HDACIs NAM (5 mM) and TSA (1 μM). Data are expressed as means ± SEMs, n = 4, ***p ≤ 0.001.

Next, we performed ubiquitin degradation assays using each of the acetyl- mimic and deacetyl-mimic forms of GKRP. As shown in Fig. 3A,B, GK robustly interacted with the WT and the K5Q GKRP acetyl-mimic, but significantly less with the K5R GKRP deacetyl-mimic (Fig. 3C,D, p ≤ 0.001). Moreover, acetylated GKRP showed increased interaction with GK *in vitro* (Supplementary Fig. 3A,B). From these results, we speculated that GKRP acetylation promotes its interaction with GK. As a result, GK-GKRP complex formation is believed to be critical for regulating GK activity and cytosolic glycolysis, consistent with a previous finding that GKRP acetylation caused GK nuclear retention^22^. To visualize whether GKRP K5 acetylation affects nuclear retention of the GKRP-GK complex, due to glucose “master sensors”, HeLa cells were incubated in 5.5 mM glucose, and immunofluorescence microscopy then performed (Fig. 3E, upper panel). As shown, most of the GK and GKRP localized to the nucleus. HeLa cells incubation in 25 mM glucose, however, resulted in the presence of both GK and GKRP in the cytosol (Fig. 3E, middle panel), consistent with other studies of this phenomenon^23^. As HDACI treatment similarly increased nuclear retention of the complex (Fig. 3E, lower panel), taken together, these results solidly suggest that GKRP acetylation increases nuclear retention of GK.

### GKRP acetylation decreases glycolytic flux

**Figure 4:**
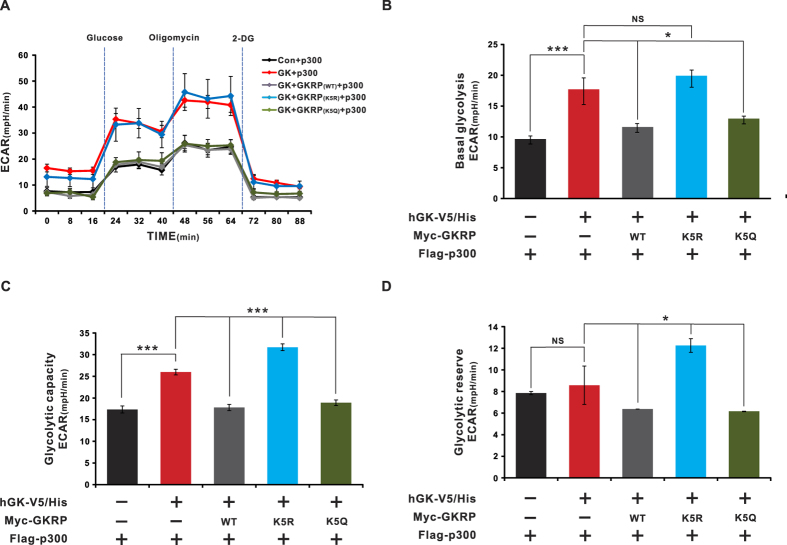
Acetylation of GKRP suppresses glycolytic flux. (**A–D**) HeLa cells were seeded in V7 cell plates at a density of 10,000 cells/well. Glycolysis assays were performed using a glycolytic stress test kit, according to the manufacturer’s protocol, on a XF24 instrument (Seahorse Biosciences). (**A**) A representative XF24 graph showing the ECAR response to glucose, oligomycin, and 2-deoxyglucose in Seahorse glucose-free medium. (**B**) Basal glycolysis calculated relative to the control after subtraction of non-glycolytic acidification. (**C**) Glycolytic capacity was calculated relative to the control following the addition of oligomycin. (**D**) Glycolytic reserve (the difference between the basal glycolysis and glycolytic capacity rates). A minimum number of n = 5 with 3–4 replicate wells per group was employed for all experiments. ECAR, extracellular acidification rate. 2-DG, 2-deoxyglucose. Con, Control. Values are expressed as means ± SEMs, *p ≤ 0.05; ***p ≤ 0.001. NS, not significant.

We next hypothesized that if GKRP acetylation increases its interaction with GK in the nucleus, cytosolic glycolysis should be reduced. Consequently, we overexpressed GK in HeLa cells and measured glycolytic flux (i.e., basal glycolysis, glycolytic capacity, and glycolytic reserve) by assessing the extracellular acidification rate (ECAR), after sequential treatment with glucose, oligomycin (an inhibitor of mitochondrial respiration), and 2-deoxyglucose (an inhibitor of glycolysis), in glucose-free Seahorse assay media (Fig. 4A and Supplementary Fig. S4A). Under those conditions, glycolytic flux significantly increased in HeLa cells overexpressing GK (Fig. 4B–D, p ≤ 0.001); that effect was negated by expression of the WT or K5Q mutant GKRP (Fig. 4B–D, p ≤ 0.05 (*) or p ≤ 0.001 (***)). In contrast, there was no significant difference in glycolytic flux in GK- or GKRP-K5R mutant-overexpressing cells (Fig. 4B–D), nor was it changed in the WT or K5Q or K5R GKRP mutants, in the absence of GK (see Supplementary Fig. S4B–D). Together, these data strongly suggest decreased glucose utilization when GKRP is acetylated.

### Increased GKRP acetylation in *db/db* mice

**Figure 5:**
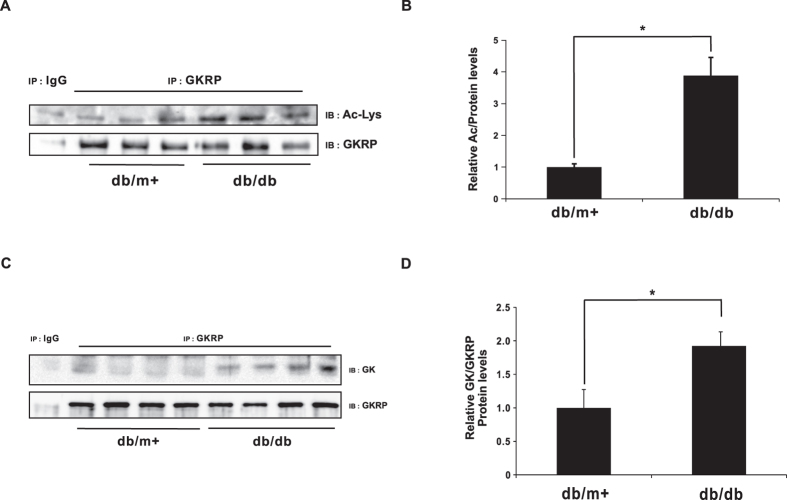
Increased acetylation of GKRP in *db/db* mice. (**A**) Western blot of acetylated GKRP in *db*/*db* mouse livers. Cell lysates were immunoprecipitated by an anti-GKRP antibody and immunoblot performed to detect endogenous acetylated GKRP using an anti-Ac-Lys antibody. (**B**) Band intensities of acetylated GKRP, as quantified by Image J software. The values from *db*/m + mice were set to 1.0 (n = 8). (**C**) Interactions between GK and GKRP in control and *db*/*db* mice. Endogenous GKRP was immunoprecipitated from liver homogenates of *db*/m + and *db*/*db* mice, using GKRP antibody, and then immunoblotted by an anti-GK antibody. (**D**) Band intensities of the GK-GKRP complexes were quantified using Image J software. The values from *db*/m + mice were set to 1.0 (n = 4). Values are expressed as means ± SEM, *p ≤ 0.05.

To assess the functional relevance of GKRP acetylation to T2DM, we performed acetylation studies in the *db/db* mouse model of diabetes, dyslipidemia, and obesity^24^. As shown in Fig. 5A,B, GKRP acetylation levels were elevated in *db/db* mice, in addition to its increased interaction with GK (Fig. 5C,D, p ≤ 0.05). These findings suggest that increasing GKRP stability and interaction with GK suppresses GK activity, thus impairing glucose homeostasis, in *db/db* mice.

### SIRT2 deacetylates GKRP

**Figure 6:**
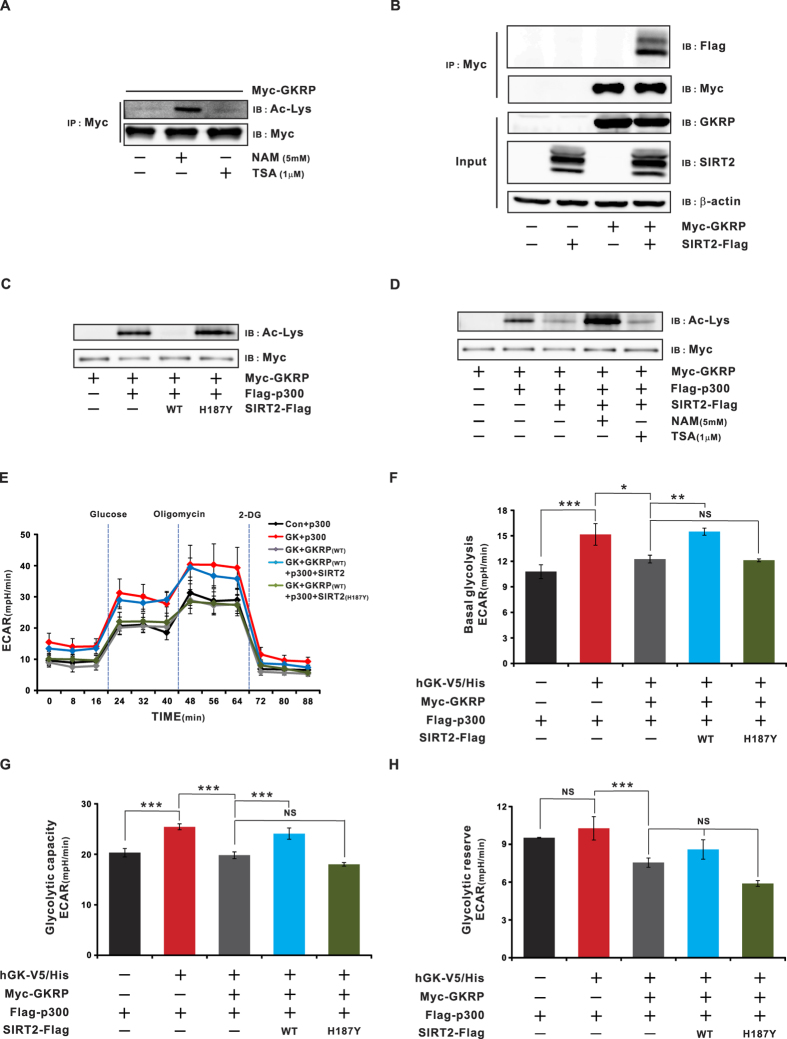
SIRT2 deacetylates GKRP. (**A**) Effect of the HDAC inhibitors NAM and TSA on GKRP acetylation. HeLa cells transfected with Myc-tagged GKRP expression vector were treated with NAM (5 mM) or TSA (1 μM) for 6 h before harvest. (**B**) Identification of interaction between SIRT2 and GKRP. HeLa cells transfected with expression plasmids of Myc-tagged GKRP and Flag-tagged SIRT2 were lysed, immunoprecipitated, and subjected to immunoblot with antibodies to Flag or Myc. (**C**) SIRT2 deacetylates GKRP in HeLa cells transfected with the indicated plasmids. Protein was precipitated with anti-Myc antibody and immunoblotted using anti-Ac-Lys or anti-Flag or anti-Myc antibodies, respectively. (**D**) GKRP deacetylation by SIRT2 is reversed by NAM. HeLa cells were transfected with the indicated plasmids, treated with NAM (5 mM) and TSA (1 μM) for 6 h, and precipitates subjected to immunoblot with antibodies to Ac, Myc or Flag. β-actin, protein level were used as an internal control. Deacetylation of GKRP regulates glycolytic flux. (E-H) HeLa cells were seeded in V7 cell plates at a density of 10,000 cells/well. Glycolysis assays were performed using a glycolytic stress test kit, according to manufacturer’s protocol, on a XF24 instrument (Seahorse Biosciences). (**E**) A representative XF24 graph showing the ECAR response to glucose, oligomycin, and 2-deoxyglucose in Seahorse glucose-free medium. (**F**) Basal glycolysis calculated relative to the control after subtraction of non-glycolytic acidification. (**G**) After the addition of oligomycin, glycolytic capacity was calculated relative to control. (**H**) Glycolytic reserve (the difference between basal glycolysis and glycolytic capacity rate). A minimum number of n = 5 with 3–4 replicate wells per group was employed for all experiments. ECAR, extracellular acidification rate. 2-DG, 2-deoxyglucose. Con, Control. SIRT2 H187Y, catalytic mutant of SIRT2. NAM, nicotinamide; TSA, Trichostatin A. Data are expressed as means ± SEMs, *p ≤ 0.05; **p ≤ 0.01; ***p ≤ 0.001. NS, not significant.

As shown in Figs 1A and 6A, GKRP acetylation increased when HeLa cells were treated with nicotinamide (NAM), an inhibitor of the NAD^+^-dependent class III (“sirtuin”) family of histone deacetylases (HDACs)^25^ (Fig. 1A). Co-immunoprecipitation assays and immunoblots further showed sirtuin 2 (SIRT2) interaction with GKRP (Fig. 6B), although other sirtuins (1 and 3–7) interacted with GKRP (see Supplementary Fig. S5A). To determine if any other sirtuins deacetylate GKRP, HeLa cells were co-tranfected with expression vectors for Myc-GKRP, various sirtuins, and p300. As shown in Supplementary Fig. S5B, GKRP was deacetylated only by SIRT2, but no other sirtuins. Further, co-overexpression of SIRT2, but not its catalytically inactive H187Y mutant, with p300, resulted in decreased deacetylation of GKRP (Fig. 6C). In addition, GKRP deacetylation by SIRT2 was reversed by NAM, but not by TSA (Fig. 6D), which does not inhibit class III HDACs. These results suggest that GKRP deacetylation is catalyzed by SIRT2.

We also assessed the effect of GKRP deacetylation on glycolytic flux, in the presence of WT or the SIRT2 inactive mutant H187Y (Fig. 6E). Under those conditions, glycolytic flux was significantly decreased when GK was co-expressed with GKRP, as compared to GK alone (Fig. 6F–H, p ≤ 0.05 and p ≤ 0.001); that effect was negated by expression of WT SIRT2 (Fig. 6F–H, p ≤ 0.01 and p ≤ 0.001). In contrast, there was no significant difference in glycolytic flux in cells overexpressing GK-GKRP or the GK-GKRP-H187Y mutant (Fig. 6F–H, p ≤ 0.05). Together, these data demonstrate increased glucose utilization when GKRP is deacetylated.

## Discussion

Post-translational modifications (PTMs) are an important means for transducing signals that “sense” metabolic changes in the body and restore homeostasis^18^. One such PTM, protein acetylation, often in concert with other PTMs, regulates metabolic enzymes^26^,^27^. However, whether acetylation impacts the metabolic effects of GK and its interacting partner, GKRP, had not yet been elucidated. This study demonstrates that GKRP Lys-5 is the principle site of acetylation by p300, as confirmed by LC-MS/MS and mutational analysis. Although Lys-5 was identified as the major acetylation site of GKRP, computational analysis predicted other acetylation sites (Supplementary Table 2). Most protein acetylation affects intracellular compartmentalization, transactivation, stability, or competition with other PTMs such as ubiquitination and phosphorylation^17^,^28^. For instance, acetylation of nuclear factor kappa-light-chain-enhancer of activated B cells (NF-κB), at distinct lysine residues, affects numerous biological processes, including transcriptional activation, DNA-binding affinity, and subcellular localization^29^. Likewise, other GKRP PTMs may yield different functional outcomes; this possibility requires further investigation. Based on these other examples of protein acetylation, we hypothesized that acetylation of GKRP could affect its biological function, a phenomena previously observed for the farnesoid X receptor (FXR) and p53, both known acetylation targets of p300^30^,^31^. Similarly, substitution of Lys (K) with Gln (Q) and Arg (R), mimicking the constitutively acetylated and deacetylated forms of p53, respectively, severely affected the various activities of that tumor suppressor, including DNA binding and gene transactivation^32^,^33^. Since non-synonymous GKRP variants have significantly different phenotypic effects^23^,^34^, it is also possible that the GKRP structural conformation may be changed by acetylation, although we did not examine this in the current study (nor do we propose its occurrence).

We also observed increased GKRP stability by inhibiting ubiquitin-proteasomal degradation during its acetylation state, as confirmed by ubiquitin and protein stability assays. In GKRP-KO mice, the stability and activity of GK is diminished, glucose levels are significantly increased, and glycemia is impaired^15^. Based on those findings, and those of our current study, we theorize that GKRP stability, regulated by acetylation, is critical to controlling homeostatic blood glucose levels.

It has been well established that GK translocates from the nucleus to the cytoplasm under conditions of high glucose^23^. Our finding, however, was that even under high glucose conditions, GK remains in the nucleus when GKRP acetylation is increased by the presence of Class III HDACIs. In addition, GK/.GKRP dimerization is decreased by ectopic expression of the deacetyl mimic of GKRP (K5R), as compared to overexpression of its WT or acetyl mimic (K5Q) forms. Indeed, glycolytic flux by GK significantly decreased in the WT, and acetyl mimic (p ≤ 0.05) GKRP forms, but was not affected by the deacetyl mimic GKRP (K5R). In consideration of GKRP activity being prominently influenced by glucose and different fructose phosphates^35^, we further propose that acetylation represents another mechanism of regulating GK activity.

Based on demonstrations that GKRP phosphorylation by AMPK inhibits both cytoplasmic translocation and activity of GK in hepatocytes^16^, we speculate that GKRP acetylation may represent a similar means of inhibiting GK activity by affecting its subcellular localization. Moreover, study of a possible interrelationship between GKRP phosphorylation and acetylation strongly warrants further investigation, akin to FOXO1 regulation by a reciprocal balance of acetylation and phosphorylation^36^.

In the presence of specific HDAC inhibitors (e.g., nicotinamide, NAM), GKRP acetylation was restored. In our experiments, NAM significantly increased acetylation, while Trichostatin A (TSA, a class I/II HDAC inhibitor) did not, suggesting that GKRP is deacetylated by the NAD^+^-dependent class III HDAC family. Among these, SIRTs 4, 6, 7, known ADP-ribosyltransferases, and SIRT5, an active desuccinylase, did not deacetylated GKRP. Among the remaining class III HDACs, only SIRT2 deacetylated GKRP (Fig. 6C and Supplementary Fig. S5B), consistent with SIRT2 involvement in deacetylation of numerous other metabolic enzymes^37^. For example, SIRT2 is believed to regulate the stability of phosphoenolypyruvate carboxykinase (PEPCK), by opposing its acetylation by p300, a critical event in the short-term regulation of gluconeogenesis^38^.

Other physiologic findings supporting our conclusions regarding increased GKRP Lys-5 acetylation were revealed in *db*/*db* diabetic/dyslipidemia mice, as hyperglycemia elevated p300-mediated protein acetylation^39^, while p300-overexpressing mice show impaired glucose clearance, with elevated blood glucose^40^. Moreover, these pathologies are similarly observed in both diabetic and GKRP-KO mice^15^,^41^. In contrast, disruption of the mouse p300 CH1-domain, a conserved protein-binding region, improves glucose tolerance and insulin sensitivity^42^, while anti-p300 adenoviral short hairpin RNAs (shRNAs) decreases hepatic glucose production^43^. Thus, GKRP acetylation likely contributes to GK’s stability and subcellular distribution, by a complicated mechanism requiring p300 and SIRT2, which subsequently affects glucose metabolism, an assumption supported by our experiments shown in Fig. 6.

Finally, hormonal effects were implicated in this phenomenon, as we observed increased GKRP acetylation in *db/db* mice, which have a high glucagon effect, but are also insulin-resistant^44^. During states of energy deficiency, glucagon induces the activity of AMPK, which phosphorylates GKRP, resulting in GK restraint to the nucleus^16^, thus suggesting that Lys 5 acetylation and GKRP phosphorylation could corroboratively control GK activity. Reduced GK activity is well-known to occur in diabetes, for which small molecule GK activators are actively being investigated^45^.

Taken together, this study strongly suggests that GKRP K5 acetylation is critical for its stability, affinity, and subcellular localization, all of which affect the short-term regulation of GK activity in the liver. A better understanding of how this process affects glycolytic flux may improve insight into possible mechanisms of metabolic pathologies such as hyperglycemia, obesity, and diabetes.

## Methods

### Plasmids and materials

Human GKRP and PCAF cDNAs were amplified by PCR from total HepG2 and HeLa cell RNA and inserted into Myc- or FLAG-tagged pSG5 (Agilent Technologies, La Jolla, CA, USA). A pSG5-based expression plasmid encoding a FLAG-tagged version of the catalytic domain of human p300 was described previously^46^. Other expression plasmids encoding FLAG-tagged hMOF and Tip60 in pcDNA3.1, and pQE30-hGK_liver-type variant 3 (v3) were kindly provided by Drs. XJ Yang (McGill University, Montreal, Canada) and D Schmoll (Sanofi-Aventis Deutschland GmbH, Germany), respectively^47^,^48^. Another expression plasmid, encoding HA-tagged ubiquitin was a kindly supplied by HG Yoon (Yonsei University College of Medicine, Republic of Korea)^49^. pcDNA3.1-hp300, pRc/RSV-mCBP-HA, and pAdEasy-Flag-hGCN5 plasmids were kind gifts from WC Greene (University of California at San Francisco, USA), RH Goodman (Oregon Health Sciences University, Portland, OR, USA), and P Puigserver (Johns Hopkins University, Baltimore, MD, USA) (Addgene plasmids #23252, #16701 and #14106), respectively^29^,^50^,^51^. DNA fragments encoding general control of amino synthesis protein 5-like 2 (GCN5) and p300 were generated by PCR from pAdEasy-Flag-hGCN5 and pcDNA3.1-hp300, respectively, and inserted into FLAG- and HA- tagged pSG5 (Agilent). V5/His-tagged hGK_v3 was derived from pQE30-hGK_liver type variant 3. A FLAG-tagged human SIRT1 (hSIRT1) expression vector was described previously^46^. Expression vectors for FLAG-tagged mouse hSIRT-2, -3, -4, -5, -6, and 7 were provided by E. Verdin (University of California, San Francisco, CA, USA)^52^. Recombinant proteins of p300 and hGK_v3 were purchased from Proteinone (Rockville, MD. USA). Cycloheximide (CHX), glucose, oligomycin, 2-deoxyglucose (2-DG), isopropyl-1-thio-β-D-galactopyranodide (IPTG), nicotinamide (NAM), trichostatin A (TSA), and C646 were purchased from Sigma-Aldrich (St. Louis, MO. USA). MG132 was purchased from Calbiochem (Darmstadt, Germany).

### Site-directed mutagenesis of plasmids

Point mutants of GKRP acetylation sites Lys 5, Lys 170, and Lys 261 were replaced by arginine or glutamine, using the QuickChange^®^ site-directed mutagenesis kit (Agilent). Oligonucleotides used for PCR are listed in Table S1.

### Cell culture and transient transfection assays

HeLa cells (CCL-2, ATCC, Manassas, VA, USA) were maintained in Dulbecco’s modified Eagle’s medium (DMEM; Hyclone, South Logan, Utah, USA) supplemented with 10% (v/v) fetal bovine serum (FBS; Hyclone), and 1% (v/v) antibiotics (Hyclone) at 37 °C, in a humidified atmosphere containing 5% CO_2_. Cells were transfected with the various expression plasmids using X-tremeGENE HP (Roche, Basel, Swizerland) at a ratio of 2:1, per the manufacturer’s protocol. After 36 h incubation, the cells were lysed for protein preparation.

### Small-interfering RNA experiments

Specific siRNA oligonucleotides targeting p300 (5′-CACCGATAACTCAGACTTGAA-3′) were synthesized by Qiagen (Venlo, Nimberg, Netherlands). The Allstars Negative Control siRNA (Qiagen) served as a negative control. HeLa cells were transfected with 10 nM siRNA using Lipofectamine RNAiMAX (Invitrogen, Carlsbad, CA, USA).

### Animal experiments

Ten nine-week-old *db*/m + (~25 g) and *db*/*db* (~35 g) male mice were purchased from Shizuoka Laboratory (Hamamatsu, Japan). Mice were housed under a 12-hr light/12-hr dark cycle and given unrestricted access to a standard chow diet and tap water. After three weeks of housing, all animals were sacrificed after the 12-hr dark cycle. Animals were cared for in accordance with the National Institutes of Health Guidelines for Animal Care. All animal experiments were approved by the Institutional Animal Care and Use Committee of the Yonsei University College of Medicine (Approval Number: 2012-0175).

### Western blot

HeLa cells were lysed with lysis buffer (20 mM HEPES [pH 7.4], 0.5% NP-40, 150 mM NaCl, 0.25% sodium deoxycholic acid, 1 mM EDTA, 1 mM phenylmethylsulfonyl fluoride [PMSF], 1 μM TSA, 5 mM NAM, and protease inhibitor cocktail [Roche, Mannheim, Germany]). The lysates were briefly vortexed and then cleared by centrifugation at 21,000 g for 20 min at 4 °C. Protein concentrations were determined using a BCA assay kit (Pierce, Rockford, IL, USA). Equal amounts of protein extracts were subjected to electrophoresis in 6–12% SDS-polyacrylamide gels and then transferred to nitrocellulose membranes (Whatman, Dassel, Germany). The membranes were blocked with milk, incubated with primary and houseradish peroxidase-conjugated secondary antibodies, and detected using SuperSignal^®^ West Pico Chemiluminescent Substrate (Thermo Fisher Scientific, Waltham, MA, USA). For *in vivo* experiments, livers were homogenized using a tissueLyser (Qiagen) for 5 min in lysis buffer, at a frequency of 25/s.

### Immunoprecipitation

HeLa cells or mouse livers were lysed, centrifuged, and pre-cleared with protein G agarose beads (Roche). Supernatants were collected and 1 μg of antibody was added. After overnight incubation, 30 μl of 50% slurry of protein-G agarose beads were added, and the samples incubated at 4 °C for 2 hr. The agarose beads were pelleted by centrifugation, washed three times with ice-cold washing buffer (20 mM HEPES [pH 7.4], 0.1% NP-40, 150 mM NaCl, 0.25% sodium deoxycholic acid, 1 mM EDTA, 1 mM PMSF, 1 μM TSA, 10 mM NAM, and protease inhibitor cocktail [Roche]), resuspended in electrophoresis sample buffer (0.09 M Tris-Cl [pH 6.8], 20% glycerol, 2% SDS, 0.1 M DTT, and 0.02% bromophenol blue), boiled for 5 min, and subjected to electrophoresis, and immunoblotting.

### Protein stability assay

Myc-tagged GKRP transfected HeLa cells were pretreated with NAM (5 mM) and TSA (1 μM) 4 h before treatment with 50 μg/ml cycloheximide (CHX) to block total protein synthesis for various times, at which GKRP protein levels were determined by western blot.

### Immunofluorescence

Immunofluorescence assays were performed as previously described^46^. Briefly, HeLa cells were transfected with GK and GKRP expression constructs. The cells were then fixed with 3.7% formaldehyde (w/v), permeabilized with 0.2% Triton X-100 in PBS, and incubated with blocking buffer containing 1% bovine serum albumin (BSA) in 0.1% PBST for 1 h at room temperature. Next, the cells were incubated with antibodies containing 1% BSA in 0.1% PBST for 12 h at 4 °C, washed with 0.1% PBST, followed by a 2 h placement in the dark with Alexa Fluor 488-conjugated donkey anti-rabbit and Alexa Fluor 568-conjugated goat anti-mouse secondary antibodies (Invitrogen). After washing with 0.1% PBST, nuclei were stained with Hoechst 33342 for 2 min, mounted using Dakocytomation Fluorescent Mounting Medium (Dako, Glostrup, Denmark), and confocal micrographs taken on an LSM 700 (Carl Zeiss, Jena, Germany), and analyzed by ZEN software (Carl Zeiss).

### Measurement of glycolytic flux using the XF24 analyzer

XF glucose flux assays were performed using an XF24 Extracellular Flux Analyzer (Seahorse Bioscience, North Billerica, MA, USA). That is based upon fluorimetric detection of O_2_ and H^+^ levels via solid-state probes on a sensor cartridge^53^. HeLa cells, seeded at 10,000 cells/well in XF24 cell plates (Seahorse Bioscience), were transfected with expression vectors for GKRP (WT), GKRP (K5R), GKRP (K5Q), GK, p300, SIRT2 (WT) and/or SIRT2 (H187Y). The following day, the media was changed to DMEM (without serum, glucose or bicarbonate, but with 2 mM glutamine), and incubated 1 h before the assay in a non-CO_2_ incubator at 37 °C. Injections of glucose (10 mM final), oligomycin (2.5 μM final) and 2-deoxyglycose (0.1 M final) were then diluted in DMEM media and loaded into ports A, B, and C sequentially. Reagents were optimized using a Glycolysis Stress Test kit (Seahorse Bioscience), using the XF24 analyzer protocol and algorithm. Each assay was run in one plate with 3–4 replicates, and repeated at least 5 times.

### Identification of modified GKRP peptide amino acids by tandem liquid chromatography-tandem mass spectrometry (LC-MS/MS)

Myc-tagged GKRP was immunoprecipitated from HeLa cells using an anti-Myc antibody. After immunoblot, protein bands were excised from stained one-dimensional electrophoresis gels and destained with 25 mM ammonium bicarbonate and 50% acetonitrile. In-gel digestion of dried gel pieces was performed using sequencing grade trypsin (Promega, Madison, WI, USA) in 25 mM ammonium bicarbonate buffer overnight at 37 °C. The tryptic peptides were desalted using a GELoader tip (Eppendorf, Hamburg, Germany) packed with 1.5 μg of POROS® 20 R2 resin (PerSpective Biosystems, Ramsey, MN, USA) and applied to a C18 RP-HPLC column (75 m × 150 mm). An Agilent 1100 Series LC system was then used to separate the trypsin-digested peptides, which were eluted with a 0–40% acetonitrile gradient for 60 min. followed by analysis using a Finnigan LCQ Deca (ThermoQuest, San Jose, CA, USA) equipped with a nanoelectrospray ion source. Spray and tube lens voltages were 1.9 kV and 40 V, respectively. The capillary temperature was maintained at 250 °C at 5 V. The individual LC-MS/MS spectra were processed using TurboSEQUEST software (ThermoQuest) and the sequences were searched in NCBI databases using MASCOT software (Matrix Science Ltd., London, UK).

### Statistical analysis

Statistics were determined using a two-tailed unpaired Student’s t test or One-Way ANOVA, using Graphpad Prism software (GraphPad, La Jolla, CA, USA). Final results we are calculated as means ± standard errors of the mean (SEMs), unless otherwise indicated. Statistical significance is represented in the figures by *p ≤ 0.05; **p ≤ 0.01; ***p ≤ 0.001.

## Additional Information

**How to cite this article**: Park, J.-M. *et al*. Acetylation of glucokinase regulatory protein decreases glucose metabolism by suppressing glucokinase activity. *Sci. Rep*. **5**, 17395; doi: 10.1038/srep17395 (2015).

## Supplementary Material

Supplementary Information

## References

[b1] Mokdad A. H. . Prevalence of obesity, diabetes, and obesity-related health risk factors, 2001. JAMA 289, 76–79 (2003). 12503980 10.1001/jama.289.1.76

[b2] Consoli A. Role of liver in pathophysiology of NIDDM. Diabetes Care 15, 430–441 (1992). 1559410 10.2337/diacare.15.3.430

[b3] Iynedjian P. B. . Tissue-specific expression of glucokinase: identification of the gene product in liver and pancreatic islets. Proc Natl Acad Sci USA 83, 1998–2001 (1986). 3515342 10.1073/pnas.83.7.1998 PMC323217

[b4] Matschinsky F. M. Glucokinase as glucose sensor and metabolic signal generator in pancreatic beta-cells and hepatocytes. Diabetes 39, 647–652 (1990). 2189759 10.2337/diab.39.6.647

[b5] Nordlie R. C., Foster J. D. & Lange A. J. Regulation of glucose production by the liver. Annu Rev Nutr 19, 379–406 (1999). 10448530 10.1146/annurev.nutr.19.1.379

[b6] Iynedjian P. B. . Transcriptional induction of glucokinase gene by insulin in cultured liver cells and its repression by the glucagon-cAMP system. J Biol Chem 264, 21824–21829 (1989). 2557341

[b7] Iynedjian P. B., Gjinovci A. & Renold A. E. Stimulation by insulin of glucokinase gene transcription in liver of diabetic rats. J Biol Chem 263, 740–744 (1988). 3275657

[b8] Kim T. H. . Interrelationship between liver X receptor alpha, sterol regulatory element-binding protein-1c, peroxisome proliferator-activated receptor gamma, and small heterodimer partner in the transcriptional regulation of glucokinase gene expression in liver. J Biol Chem 284, 15071–15083 (2009). 19366697 10.1074/jbc.M109.006742 PMC2685689

[b9] Yoshida K. . Pancreatic glucokinase is activated by insulin-like growth factor-I. Endocrinology 148, 2904–2913 (2007). 17317782 10.1210/en.2006-1149

[b10] Beer N. L. . The P446L variant in GCKR associated with fasting plasma glucose and triglyceride levels exerts its effect through increased glucokinase activity in liver. Hum Mol Genet 18, 4081–4088 (2009). 19643913 10.1093/hmg/ddp357 PMC2758140

[b11] Van Schaftingen E., Detheux M. & Veiga da Cunha M. Short-term control of glucokinase activity: role of a regulatory protein. FASEB J 8, 414–419 (1994). 8168691 10.1096/fasebj.8.6.8168691

[b12] de la Iglesia N. . Glucokinase regulatory protein is essential for the proper subcellular localisation of liver glucokinase. FEBS Lett 456, 332–338 (1999). 10456334 10.1016/s0014-5793(99)00971-0

[b13] Agius L. & Stubbs M. Investigation of the mechanism by which glucose analogues cause translocation of glucokinase in hepatocytes: evidence for two glucose binding sites. Biochem J 346 **Pt 2**, 413–421 (2000). 10677361 PMC1220868

[b14] Detheux M., Vandercammen A. & Van Schaftingen E. Effectors of the regulatory protein acting on liver glucokinase: a kinetic investigation. Eur J Biochem 200, 553–561 (1991). 1889418 10.1111/j.1432-1033.1991.tb16218.x

[b15] Farrelly D. . Mice mutant for glucokinase regulatory protein exhibit decreased liver glucokinase: a sequestration mechanism in metabolic regulation. Proc Natl Acad Sci USA 96, 14511–14516 (1999). 10588736 10.1073/pnas.96.25.14511 PMC24467

[b16] Mukhtar M. H. . Inhibition of glucokinase translocation by AMP-activated protein kinase is associated with phosphorylation of both GKRP and 6-phosphofructo-2-kinase/fructose-2,6-bisphosphatase. Am J Physiol Regul Integr Comp Physiol 294, R766–774 (2008). 18199594 10.1152/ajpregu.00593.2007

[b17] Zhao Y., Wang Y. & Zhu W. G. Applications of post-translational modifications of FoxO family proteins in biological functions. J Mol Cell Biol 3, 276–282 (2011). 21669942 10.1093/jmcb/mjr013

[b18] Guan K. L. & Xiong Y. Regulation of intermediary metabolism by protein acetylation. Trends Biochem Sci 36, 108–116 (2011). 20934340 10.1016/j.tibs.2010.09.003 PMC3038179

[b19] Xu W. S., Parmigiani R. B. & Marks P. A. Histone deacetylase inhibitors: molecular mechanisms of action. Oncogene 26, 5541–5552 (2007). 17694093 10.1038/sj.onc.1210620

[b20] Bowers E. M. . Virtual ligand screening of the p300/CBP histone acetyltransferase: identification of a selective small molecule inhibitor. Chem Biol 17, 471–482 (2010). 20534345 10.1016/j.chembiol.2010.03.006 PMC2884008

[b21] Li A. . Prediction of Nepsilon-acetylation on internal lysines implemented in Bayesian Discriminant Method. Biochem Biophys Res Commun 350, 818–824 (2006). 17045240 10.1016/j.bbrc.2006.08.199 PMC2093955

[b22] Shiota C. . Nuclear import of hepatic glucokinase depends upon glucokinase regulatory protein, whereas export is due to a nuclear export signal sequence in glucokinase. J Biol Chem 274, 37125–37130 (1999). 10601273 10.1074/jbc.274.52.37125

[b23] Rees M. G. . Cellular characterisation of the GCKR P446L variant associated with type 2 diabetes risk. Diabetologia 55, 114–122 (2012). 22038520 10.1007/s00125-011-2348-5 PMC3276843

[b24] Kobayashi K. . The db/db mouse, a model for diabetic dyslipidemia: molecular characterization and effects of Western diet feeding. Metabolism 49, 22–31 (2000). 10647060 10.1016/s0026-0495(00)90588-2

[b25] Avalos J. L., Bever K. M. & Wolberger C. Mechanism of sirtuin inhibition by nicotinamide: altering the NAD(+) cosubstrate specificity of a Sir2 enzyme. Mol Cell 17, 855–868 (2005). 15780941 10.1016/j.molcel.2005.02.022

[b26] Glozak M. A., Sengupta N., Zhang X. & Seto E. Acetylation and deacetylation of non-histone proteins. Gene 363, 15–23 (2005). 16289629 10.1016/j.gene.2005.09.010

[b27] Zhao S. . Regulation of cellular metabolism by protein lysine acetylation. Science 327, 1000–1004 (2010). 20167786 10.1126/science.1179689 PMC3232675

[b28] Dai C. & Gu W. p53 post-translational modification: deregulated in tumorigenesis. Trends Mol Med 16, 528–536 (2010). 20932800 10.1016/j.molmed.2010.09.002 PMC2978905

[b29] Chen L. F., Mu Y. & Greene W. C. Acetylation of RelA at discrete sites regulates distinct nuclear functions of NF-kappaB. EMBO J 21, 6539–6548 (2002). 12456660 10.1093/emboj/cdf660 PMC136963

[b30] Kemper J. K. . FXR acetylation is normally dynamically regulated by p300 and SIRT1 but constitutively elevated in metabolic disease states. Cell Metab 10, 392–404 (2009). 19883617 10.1016/j.cmet.2009.09.009 PMC2785075

[b31] Li M., Luo J., Brooks C. L. & Gu W. Acetylation of p53 inhibits its ubiquitination by Mdm2. J Biol Chem 277, 50607–50611 (2002). 12421820 10.1074/jbc.C200578200

[b32] Wang Y. H. . Identification and characterization of a novel p300-mediated p53 acetylation site, lysine 305. J Biol Chem 278, 25568–25576 (2003). 12724314 10.1074/jbc.M212574200

[b33] Feng L. . Functional analysis of the roles of posttranslational modifications at the p53 C terminus in regulating p53 stability and activity. Mol Cell Biol 25, 5389–5395 (2005). 15964796 10.1128/MCB.25.13.5389-5395.2005 PMC1157004

[b34] Rees M. G. . Correlation of rare coding variants in the gene encoding human glucokinase regulatory protein with phenotypic, cellular, and kinetic outcomes. J Clin Invest 122, 205–217 (2012). 22182842 10.1172/JCI46425 PMC3248284

[b35] Zelent B. . Analysis of the co-operative interaction between the allosterically regulated proteins GK and GKRP using tryptophan fluorescence. Biochem J 459, 551–564 (2014). 24568320 10.1042/BJ20131363 PMC4109836

[b36] Qiang L., Banks A. S. & Accili D. Uncoupling of acetylation from phosphorylation regulates FoxO1 function independent of its subcellular localization. J Biol Chem 285, 27396–27401 (2010). 20519497 10.1074/jbc.M110.140228 PMC2930737

[b37] Schwer B. & Verdin E. Conserved metabolic regulatory functions of sirtuins. Cell Metab 7, 104–112 (2008). 18249170 10.1016/j.cmet.2007.11.006

[b38] Jiang W. . Acetylation regulates gluconeogenesis by promoting PEPCK1 degradation via recruiting the UBR5 ubiquitin ligase. Mol Cell 43, 33–44 (2011). 21726808 10.1016/j.molcel.2011.04.028 PMC3962309

[b39] Chen S. . Transcriptional coactivator p300 regulates glucose-induced gene expression in endothelial cells. Am J Physiol Endocrinol Metab 298, E127–137 (2010). 19903865 10.1152/ajpendo.00432.2009

[b40] Bricambert J. . Salt-inducible kinase 2 links transcriptional coactivator p300 phosphorylation to the prevention of ChREBP-dependent hepatic steatosis in mice. J Clin Invest 120, 4316–4331 (2010). 21084751 10.1172/JCI41624 PMC2993582

[b41] Brozinick J. T. Jr. . GLUT4 overexpression in db/db mice dose-dependently ameliorates diabetes but is not a lifelong cure. Diabetes 50, 593–600 (2001). 11246879 10.2337/diabetes.50.3.593

[b42] Bedford D. C. . Disrupting the CH1 domain structure in the acetyltransferases CBP and p300 results in lean mice with increased metabolic control. Cell Metab 14, 219–230 (2011). 21803292 10.1016/j.cmet.2011.06.010 PMC3163393

[b43] He L. . Activation of basal gluconeogenesis by coactivator p300 maintains hepatic glycogen storage. Mol Endocrinol 27, 1322–1332 (2013). 23770612 10.1210/me.2012-1413 PMC3725339

[b44] Hagberg C. E. . Targeting VEGF-B as a novel treatment for insulin resistance and type 2 diabetes. Nature 490, 426–430 (2012). 23023133 10.1038/nature11464

[b45] Matschinsky F. M. Assessing the potential of glucokinase activators in diabetes therapy. Nat Rev Drug Discov 8, 399–416 (2009). 19373249 10.1038/nrd2850

[b46] Park J. M. . Role of resveratrol in FOXO1-mediated gluconeogenic gene expression in the liver. Biochem Biophys Res Commun 403, 329–334 (2010). 21078299 10.1016/j.bbrc.2010.11.028

[b47] Anderka O. . Biophysical characterization of the interaction between hepatic glucokinase and its regulatory protein: impact of physiological and pharmacological effectors. J Biol Chem 283, 31333–31340 (2008). 18809676 10.1074/jbc.M805434200

[b48] Ullah M. . Molecular architecture of quartet MOZ/MORF histone acetyltransferase complexes. Mol Cell Biol 28, 6828–6843 (2008). 18794358 10.1128/MCB.01297-08 PMC2573306

[b49] Yoo J. Y. . Nuclear hormone receptor corepressor promotes esophageal cancer cell invasion by transcriptional repression of interferon-gamma-inducible protein 10 in a casein kinase 2-dependent manner. Mol Biol Cell 23, 2943–2954 (2012). 22675025 10.1091/mbc.E11-11-0947 PMC3408420

[b50] Chrivia J. C. . Phosphorylated CREB binds specifically to the nuclear protein CBP. Nature 365, 855–859 (1993). 8413673 10.1038/365855a0

[b51] Lerin C. . GCN5 acetyltransferase complex controls glucose metabolism through transcriptional repression of PGC-1alpha. Cell Metab 3, 429–438 (2006). 16753578 10.1016/j.cmet.2006.04.013

[b52] Schwer B. . Reversible lysine acetylation controls the activity of the mitochondrial enzyme acetyl-CoA synthetase 2. Proc Natl Acad Sci USA 103, 10224–10229 (2006). 16788062 10.1073/pnas.0603968103 PMC1502439

[b53] Wu M. . Multiparameter metabolic analysis reveals a close link between attenuated mitochondrial bioenergetic function and enhanced glycolysis dependency in human tumor cells. Am J Physiol Cell Physiol 292, C125–136 (2007). 16971499 10.1152/ajpcell.00247.2006

