# Supplementary material for: Acetylation of glucokinase regulatory protein decreases glucose metabolism by suppressing glucokinase activity
Source: Sci Rep. 2015 Dec 1;5:17395. doi: 10.1038/srep17395 (PMC4664969; doi:10.1038/srep17395)

**Supplementary Information**

**Acetylation of glucokinase regulatory protein decreases glucose metabolism by suppressing glucokinase activity**

Joo-Man Park^1^, Tae-Hyun Kim^1^, Seong-Ho Jo^1^, Mi-Young Kim^1^, Yong-Ho Ahn^1,2,#^

^1^Department of Biochemistry and Molecular Biology, ^2^Brain Korea 21 PLUS Project for Medical Sciences, Yonsei University College of Medicine, 50-1 Yonsei-ro, Seodaemun-gu, Seoul 120-752, Republic of Korea

^#^Address correspondence to: Yong-Ho Ahn, MD, PhD, Dept. of Biochemistry and Molecular Biology, Yonsei Universirty College of Medicine, 50-1 Yonsei-ro, Seodaemun-gu, Seoul 120-752, Republic of Korea. Tel: +82-2-2228-0835, Fax: +82-2-312-5041 E-mail:yha111@yuhs.ac

**Supplementary Methods**

**Total RNA extraction, reverse transcription, and quantitative real time PCR (qPCR).** Total RNA was extracted using Easy-spin RNA extraction kits (iNtRON, Kyeonggi-do, Republic of Korea), according to the manufacturer’s protocol. Tissue samples were homogenized in Easy-Spin Lysis buffer (1 ml /100 mg of tissue; iNtRON) using a TissueLyser (Qiagen, Venlo, Nimberg, Netherlands) for 5 min at a frequency of 25/s. cDNA was generated from 2.5 μg total RNA using GoScript™ (Promega, Madison, WI, USA), and qPCR was performed using the StepOne™ Sequence Detection System (Applied Biosystems, Foster City, CA, USA), according to the manufacturer’s protocol. Gene mRNA levels were expressed as folds-increase relative to basal transcription levels, using the comparative C_T_ method. The amount of target mRNA was normalized to 18S mRNA levels.

**Expression and Purification of Glucokinase Regulatory Protein.** To prepare bacterial recombinant fusion proteins, the cDNA of human GKRP was ligated into pGEXT-4T1 and transformed into *E. coli* stain BL21 (DE3) competent cells. Bacteria cultures were incubated at 37 ^o^C with shaking for 12 h (usually an OD_600_ of at least 0.6 was reached), and then 0.1mM isopropyl-1-thio-β-D-galactopyranodide (IPTG) was added. After incubation for another 4 h at 30^o^C, the bacteria were pelleted by centrifugation and the pellet was stored at -80^o^C until use. Purification of glutathione S-transferase (GST) fusion proteins was carried out using glutathione-Sepharose 4 Fast Flow beads (GE Healthcare, Uppsala, Sweden) for 4 h. following the manufacturer’s protocol.

***In vitro* acetyltransferase assay.** Substrate proteins (GST and GST-GKRP) were incubation at 30^o^C for 90 min in 30μl with 5ＸHAT assay buffer (250mM Tris [pH 8.0], 25% glycerol, 0.5mM EDTA, 250mM KCl, 50mM sodium butyrate, 5mM trichostatin A (TSA), 10mM Nicotinamide (NAM), and 10mM acetyl-CoA) and acetyltransferase enzyme (recombinant p300, Proteinone, Rockville, MD. USA). For western-blotting, the reactions were stopped by adding electrophoresis sample buffer (0.09 M Tris-Cl [pH 6.8], 20% glycerol, 2% SDS, 0.1M DTT, and 0.02% bromophenol blue) after incubation and then, boiled for 5 min, and subjected to electrophoresis, and immunoblotting.


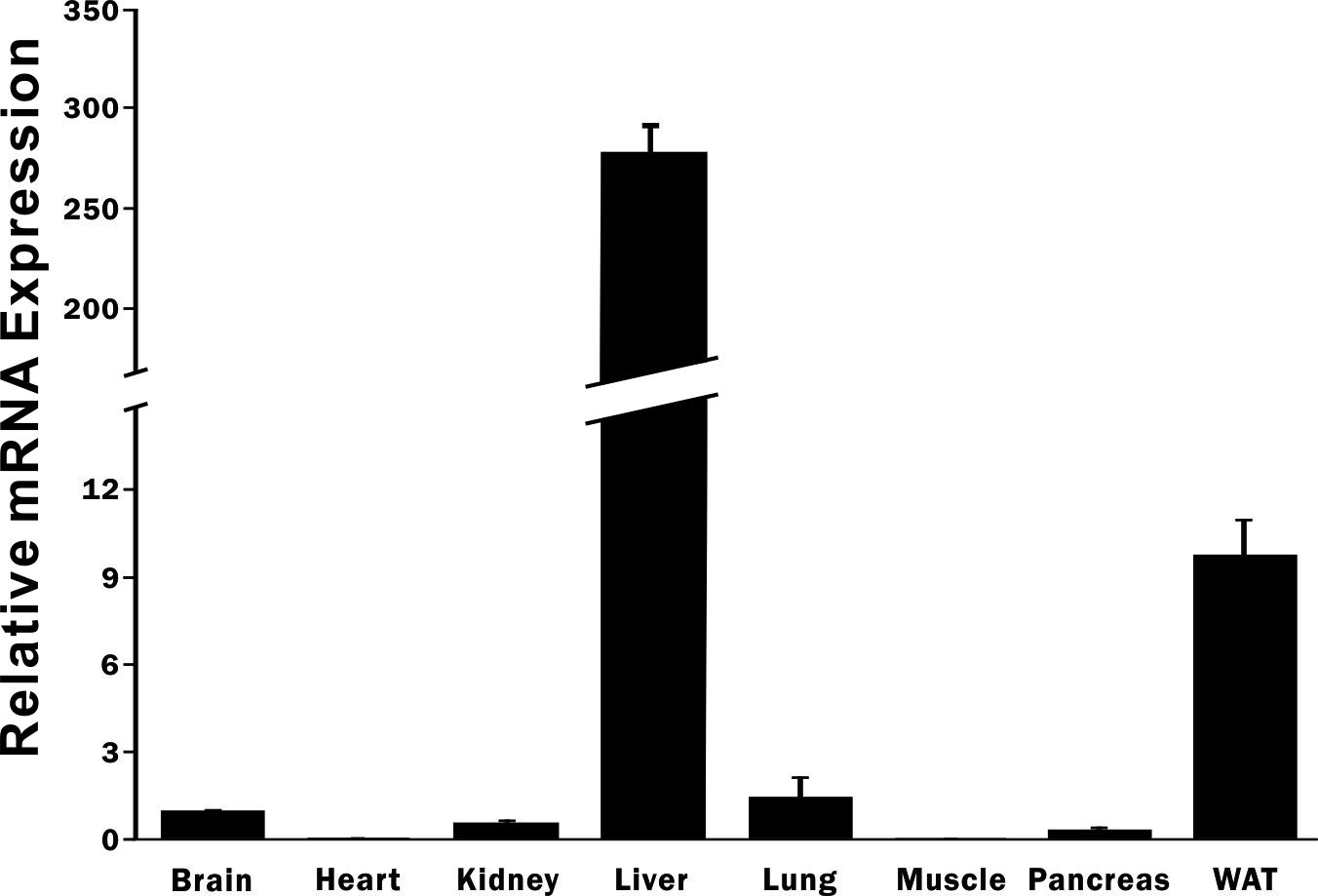


**Figure S1.** Tissue distribution of mouse Gckr. Each tissue was isolated from C57BL/6J mice. Gckr mRNA levels of were measured by qPCR as described in the Materials and Methods. The quantity of mRNA was normalized to 18s mRNA. Data were processed by the comparative C_T_ method and expressed as folds-increase relative to the brain transcription level. Results are presented as means ± SE of three independent experiments in triplicates. WAT, white adipose tissue.


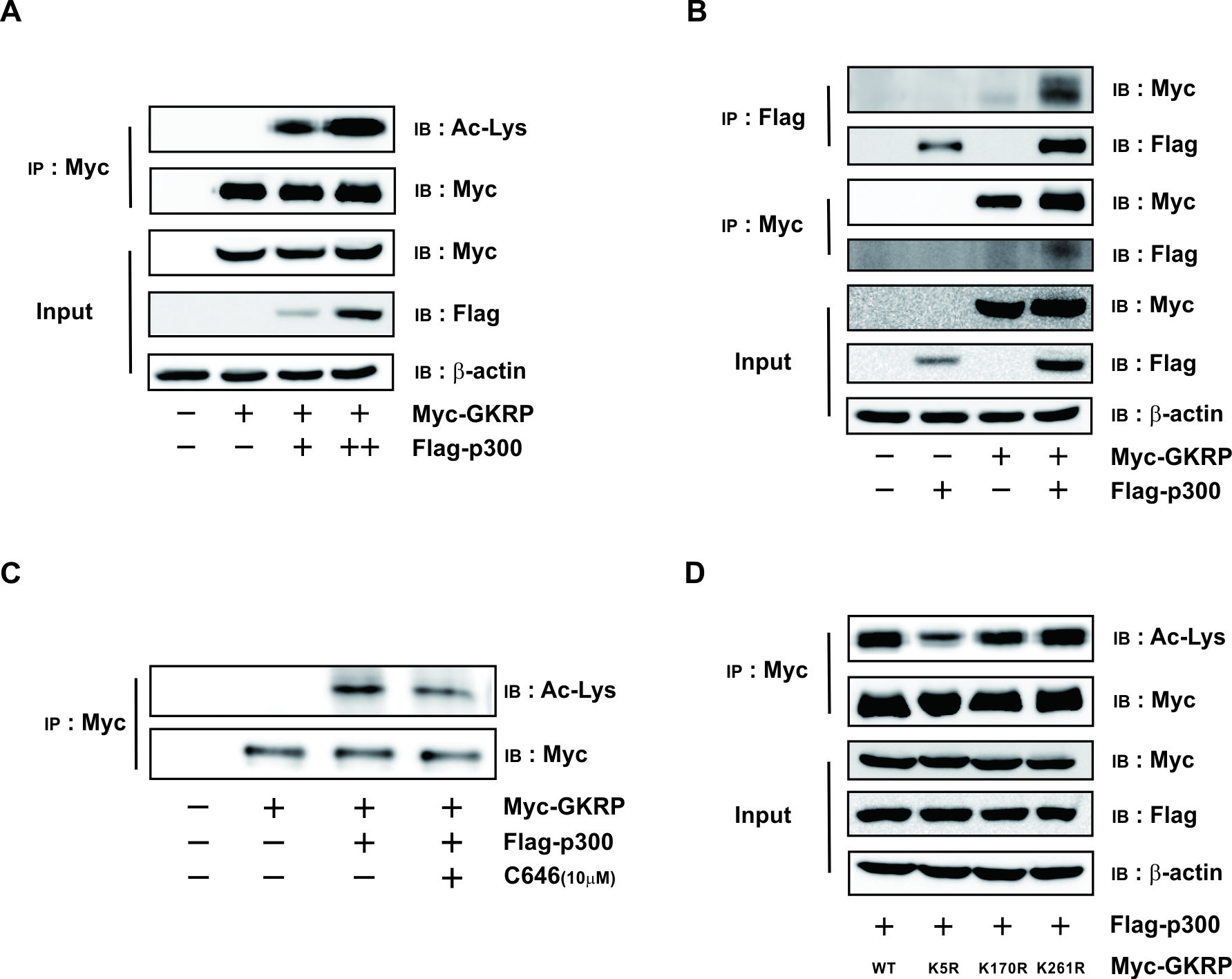


**Figure S2.** GKRP is acetylated by p300. (A) GKRP is acetylated by p300 in a dose-dependent manner. Myc-tagged GKRP (2μg) and pSG5-Flag catalytic domain of p300 (3, 6μg) were co-expressed in HeLa cells and precipitated with anti-Myc antibody. (B) Acetylation of GKRP by p300 occurs through direct interaction. HeLa cells were transfected with Myc-tagged GKRP and Flag tagged catalytic domain of p300 and purified with either anti-Myc or anti-Flag antibody. (C) Acetylation of GKRP is inhibited by C646. HeLa cells were transfected with Myc-tagged GKRP and Flag tagged catalytic domain of p300 in the presence or absence of 10mM C646, p300 specific inhibitor, and purified with either anti-Myc antibody. (D) Effect of site-specific mutation on the potential acetylation sites of GKRP. Substitutions of Lys (K) with Arg (R) at the indicated sites are shown in parenthesis. HeLa cells were transfected with the indicated mutants or wild type plasmid and precipitated with an anti-Myc antibody. Acetylated GKRP was detected by anti-Ac-Lys antibodies. β-actin was used as an internal control.


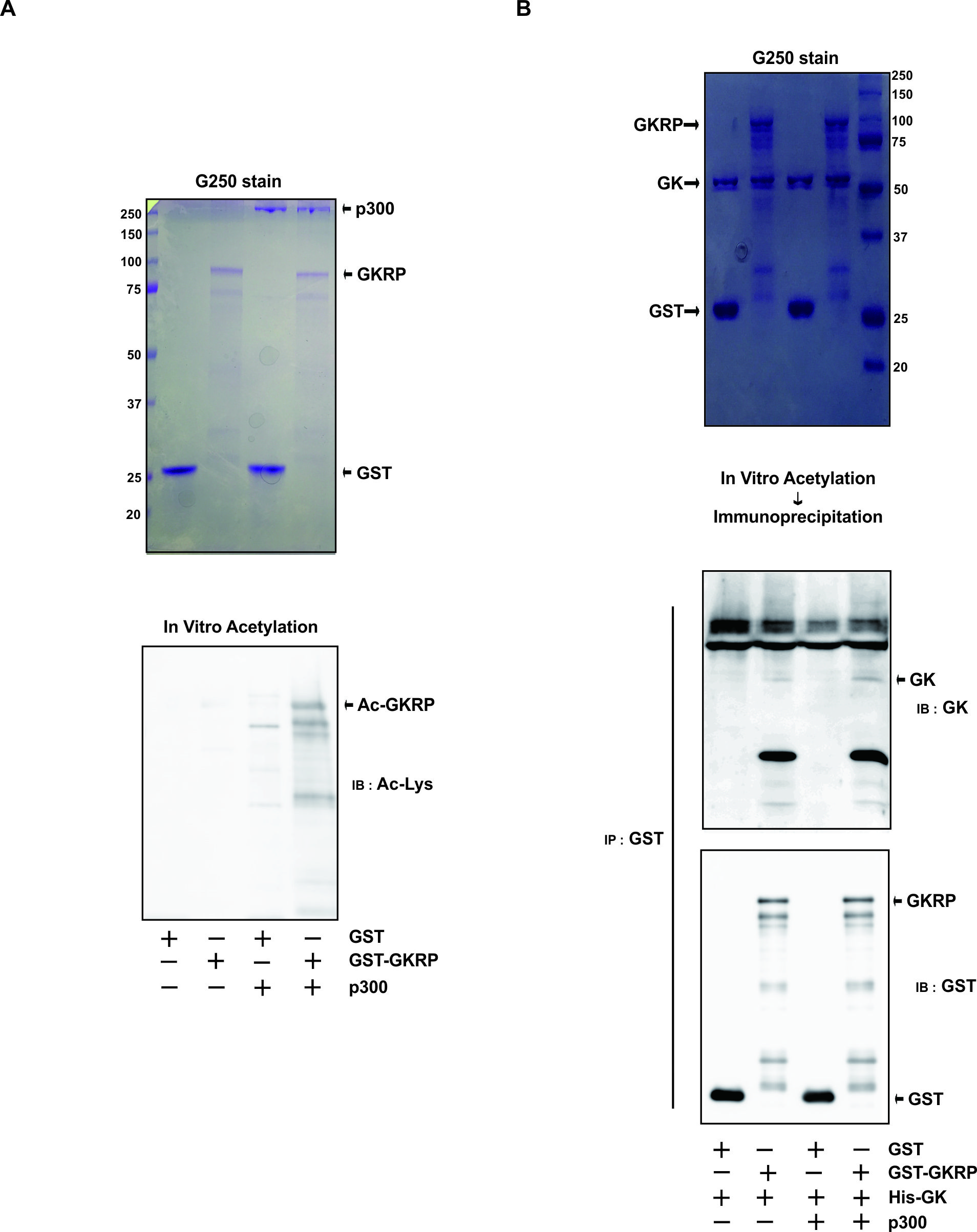


**Figure S3.** GKRP acetylation regulates the interaction with GK. (A) *In vitro* GKRP acetylation. Top panel shows the coomassie brilliant blue (CBB)-stained SDS-polyacrylamide gel. Lower panel shows that GST or GST-GKRP was incubated with the presence or absence of p300 by *in vitro* acetylation assay. After incubation, the reaction mixture was resolved by SDS-PAGE and acetylated GKRP was detected by anti-Ac-Lys antibodies. (B) *In vitro* GKRP-GK complex. Top panel shows the CBB-stained SDS-PAGE gel. Middle panel displays the experimental outline. Lower panel shows that in vitro acetylation reacted mixture and recombinant GK were precipitated with anti-GST antibody, and blotted with an anti-GK antibody.


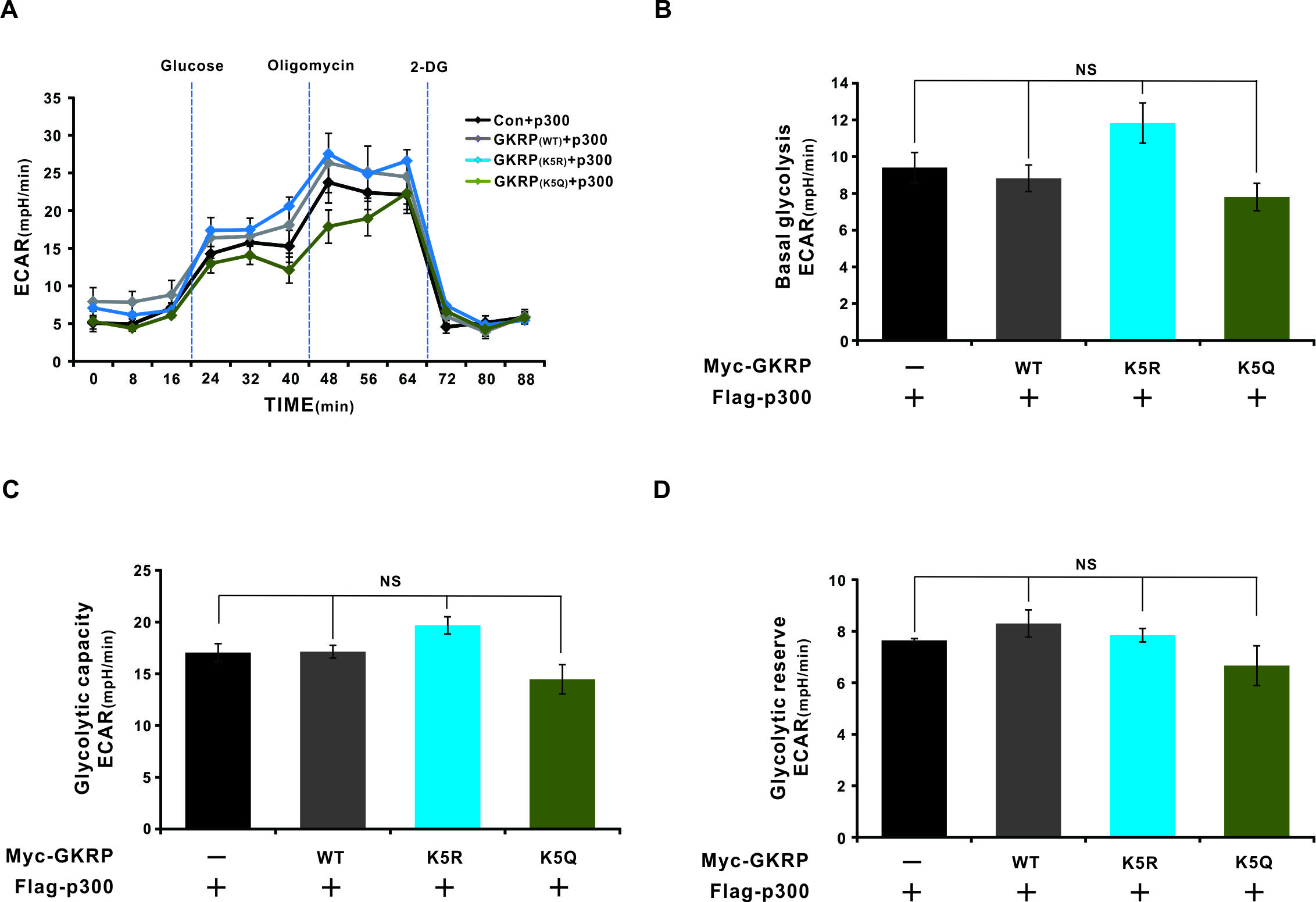


**Figure S4.** Glycolytic flux is not changed by mutant forms of GKRP in the absence of GK. HeLa cells were seeded in V7 cell plates at a density of 10,000cells/well. Glycolysis assays were performed using glycolytic stress test kits, according to manufacture’s protocol using XF24 instrument (Seahorse Biosciences). (A) A representative XF24 output graph showing the ECAR response to glucose, oligomycin and 2-DG in Seahorse glucose-free medium. (B) Basal glycolysis calculated relative to the control after subtraction of the non-glycolytic acidification. (C) After addition of oligomycin, glycolytic capacity was calculated relative to control. (D) Levels of glycolytic reserve, i.e., the difference between basal glycolysis and glycolytic capacity rate. The minimum number of n = 5 with 3–4 replicate wells per group was employed for all experiments. K5R, deacetylated mimic GKRP; K5Q, acetylated mimic GKRP. ECAR, extracellular acidification rate. 2-DG, 2-deoxyglucose. Con, Control. Values are expressed as mean ± SEM. NS, not significant.


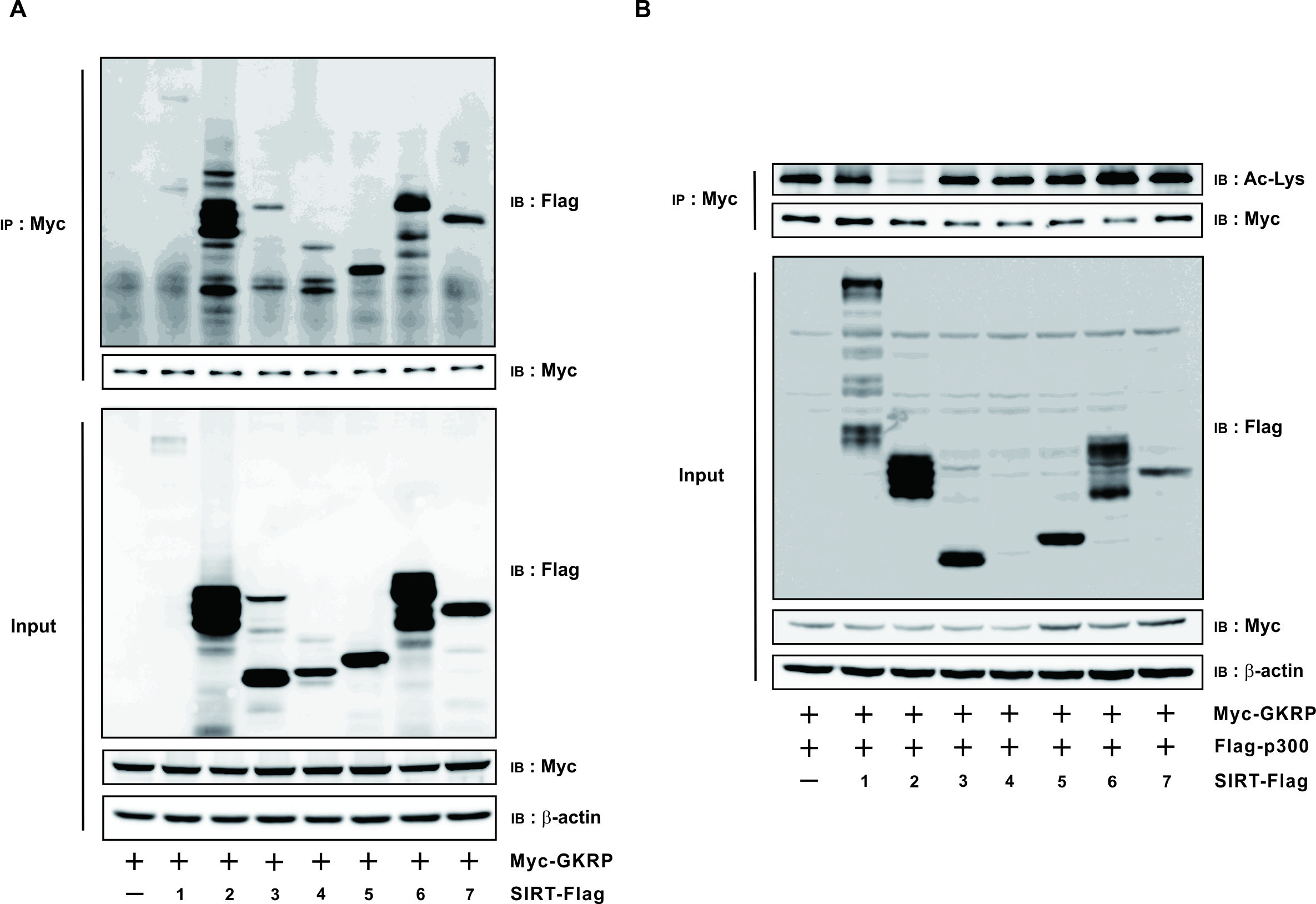


**Figure S5.** Deacetylation of GKRP by SIRT2. (A) Interaction of various SIRT family members with GKRP. HeLa cells were transfected with expression plasmids of Myc-tagged GKRP and various SIRTs. Proteins were precipitated with anti-Myc antibody and detected with anti-Flag or anti-Myc antibody. (B) GKRP deacetylation occurs by SIRT2. HeLa cells were transfected with expression plasmids of Myc-tagged GKRP and various SIRTs. Proteins were precipitated with anti-Myc antibody. Acetylated GKRP was detected by anti-Ac-Lys antibodies.

**Table S1.** Sequences of oligonucleotides used for generating site-directed of GKRP and qPCR.


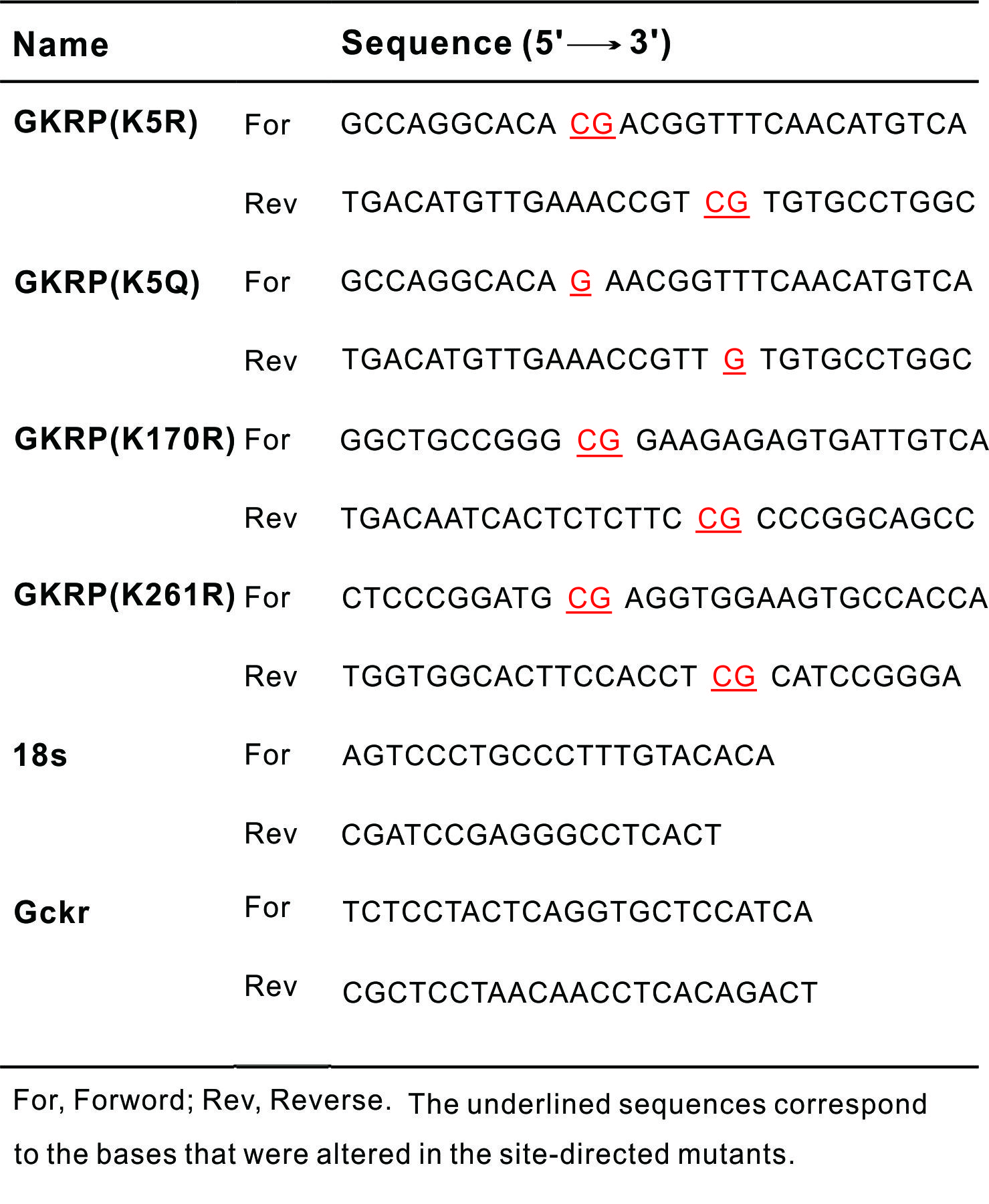


**Table S2.** Prediction of potential acetylation sites using Prediction of Acetylation on Internal Lysines (PAIL) program.


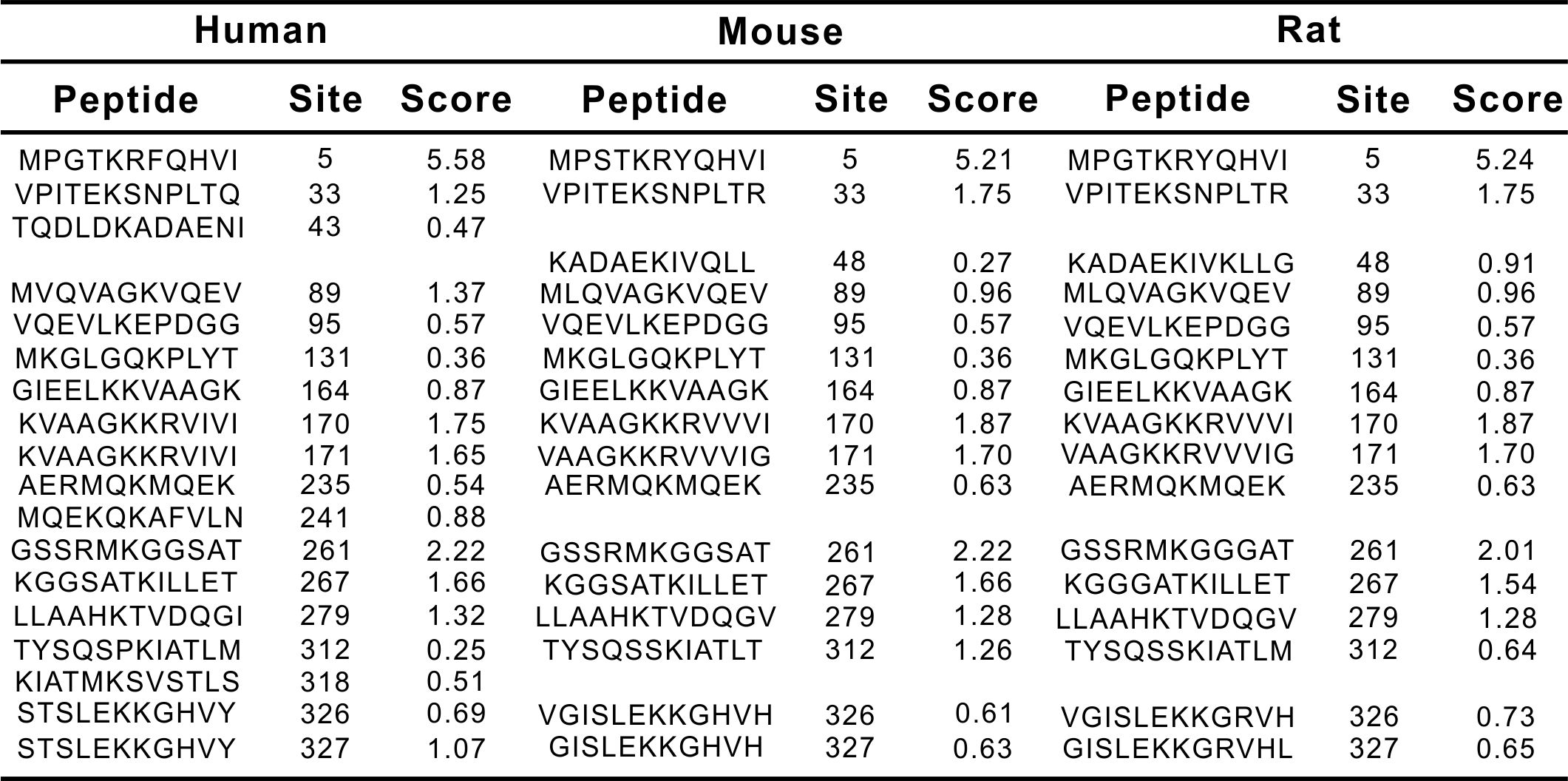


**Table S3. List of antibodies used in this study.**


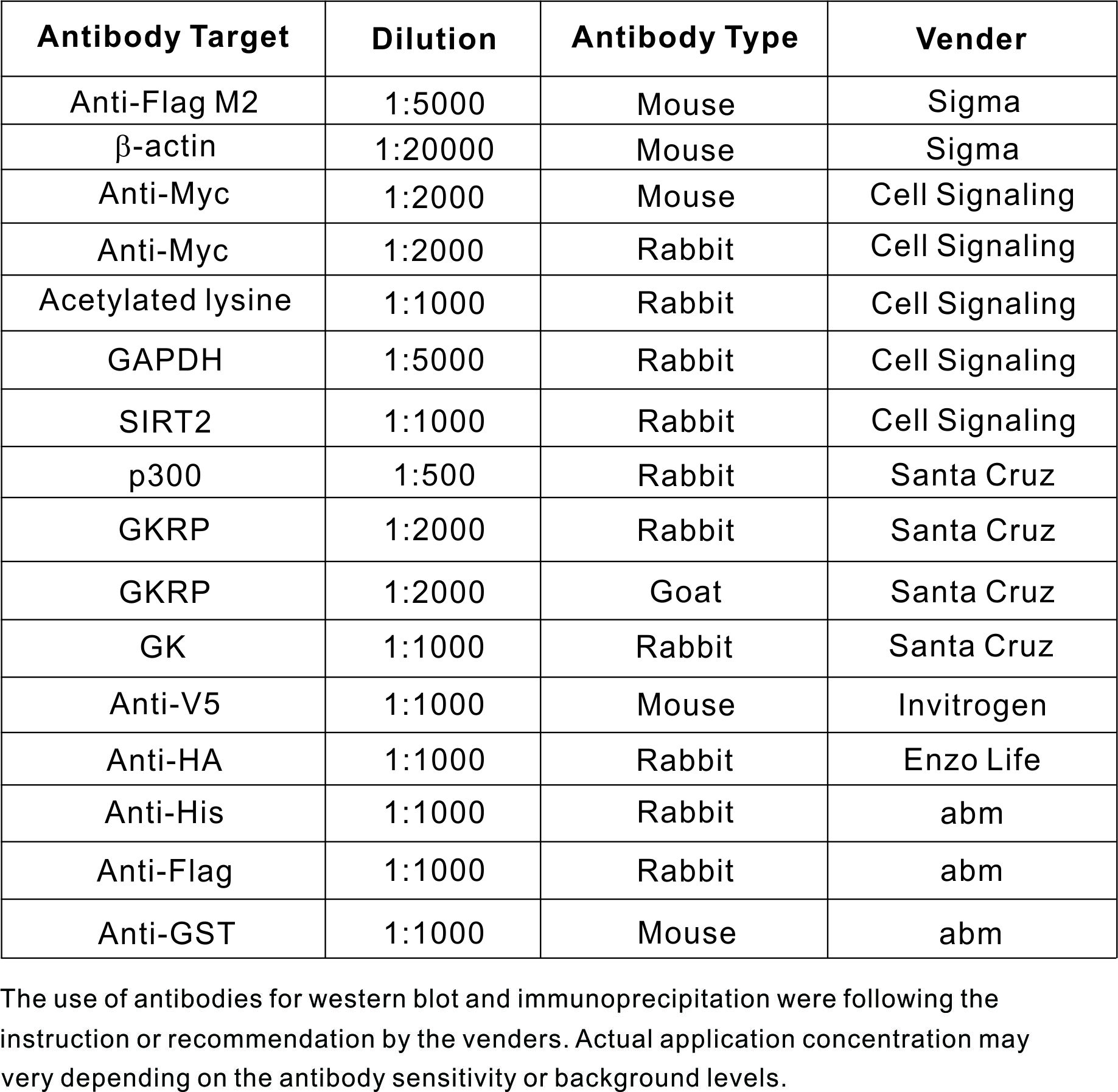

Supplement: Supplementary Information [file srep17395-s1.doc]
